# A simple and cost‐effective method for studying anoxia tolerance in plants

**DOI:** 10.1002/aps3.11509

**Published:** 2023-01-28

**Authors:** Orla L. Sherwood, Rebecca Carroll, Stephen Burke, Paul F. McCabe, Joanna Kacprzyk

**Affiliations:** ^1^ School of Biology and Environmental Science University College Dublin Dublin Ireland

**Keywords:** abiotic stress, anoxia tolerance, *Arabidopsis*, barley

## Abstract

**Premise:**

We developed a novel, cost‐effective protocol that facilitates testing anoxia tolerance in plants without access to specialized equipment.

**Methods and Results:**

*Arabidopsis thaliana* and barley (*Hordeum vulgare*) seedlings were treated in airtight 2‐L Kilner jars. An anoxic atmosphere was generated using Oxoid AnaeroGen 2.5‐L sachets placed on in‐house, custom‐built wire stands. The performed experiments confirmed a higher sensitivity to low oxygen stress previously observed in *anac017 A. thaliana* mutants and the positive effect of exogenous sucrose on anoxia tolerance reported by previous studies in *A. thaliana*. Barley seedlings displayed typical responses to anoxia treatment, including shoot growth cessation and the induction of marker genes for anaerobic metabolism and ethylene biosynthesis in root tissue.

**Conclusions:**

The results validate the novel method as an inexpensive, simple alternative for testing anoxia tolerance in plants, where access to an anaerobic workstation is not possible. The novel protocol requires minimum investment and is easily adaptable.

Oxygen deficiency (hypoxia) and oxygen absence (anoxia) are abiotic stresses with dramatic effects on plant physiology. They result in an “energy crisis” in plant cells and tissues due to restricted respiration (Gibbs and Greenway, 2003; Huang et al., 2008). The ability to cope with hypoxia and/or anoxia is particularly relevant in the context of plant flooding tolerance due to the relatively low solubility of oxygen in water and the slow diffusion of gases in water compared to air (Jackson, 1985; Voesen et al., 2006). Depending on the height of the water column produced, the extent of flooding may range from soil waterlogging to complete submergence, when the aerial plant tissues are covered by water (Jackson and Colmer, 2005). Furthermore, the adverse effects of these stresses can be exacerbated by ice encasement, where the water flooding the field freezes and the rate of exchange of respiratory gases drops to extremely low levels, requiring anoxia tolerance for plant survival (Andrews, 1996). Indeed, a recent meta‐analysis estimated that flooding‐induced global crop yield losses may exceed 30% (Tian et al., 2021), and this is projected to increase in many regions due to increasingly volatile weather associated with climate change (Hov et al., 2013; Lehtonen et al., 2014). Consequently, there is an urgent need to further elucidate the molecular mechanisms that underpin plant responses to oxygen deprivation (Zahra et al., 2021) and develop flood‐resilient cultivars.

Most studies of anoxia tolerance in the model species *Arabidopsis thaliana* (L.) Heynh. have been performed using an enclosed anaerobic workstation to provide an oxygen‐free environment for plant incubation, such as the Anaerobic System Model 1025 (Forma Scientific, Marietta, Ohio, USA) or 1 Person Hypoxic Glove Box (Coy Laboratory Products, Grass Lake, Michigan, USA) (Kürsteiner et al., 2003; Banti et al., 2008; Tagliani et al., 2020). A similar setup was also used to study anoxia tolerance in rice seedlings (Lasanthi‐Kudahettige et al., 2007). However, not all laboratories have access to a commercial anaerobic workstation, and purchasing one is a significant investment (in excess of €20,000, dependent on specifications) that may be unaffordable to many research groups. An alternative approach reported in the literature involves placing plants in airtight jars flushed with a nitrogen gas stream and/or premixed air (Konkina et al., 2021). However, this also requires access to anaerobic jars with gas valves or another self‐contained anaerobic system, and thus may incur significant costs.

Herein, we describe and validate a novel airtight jar method for testing anoxia tolerance in plants that is reliable, cost‐effective, and easily accessible. Airtight Kilner jars were purchased from a local household and kitchen supplies store (less than €10 per 2‐L jar) and used in combination with Oxoid AnaeroGen 2.5‐L sachets (Thermo Fisher Scientific, Basingstoke, United Kingdom), an affordable atmosphere‐generating product frequently used by microbiologists for the cultivation of anaerobic bacteria (~€60 per pack of 10 sachets, depending on the geographic location/supplier). We successfully used the protocol to corroborate findings previously obtained for *A. thaliana* with other methods, such as use of anaerobic workstations and submergence tolerance testing (Banti et al., 2008; Bui et al., 2020; Meng et al., 2020). This suggests that the developed method can be successfully used to dissect the mechanisms underlying anoxia tolerance in this model species, where a multitude of genetic resources are readily available. Additionally, we demonstrate the suitability of the method for inducing anoxia responses in barley (*Hordeum vulgare* L.) seedlings and propose that it can be applied to identify genotypes and cultivars resilient to this abiotic stress.

## METHODS AND RESULTS

### Experimental setup

**Figure 1:**
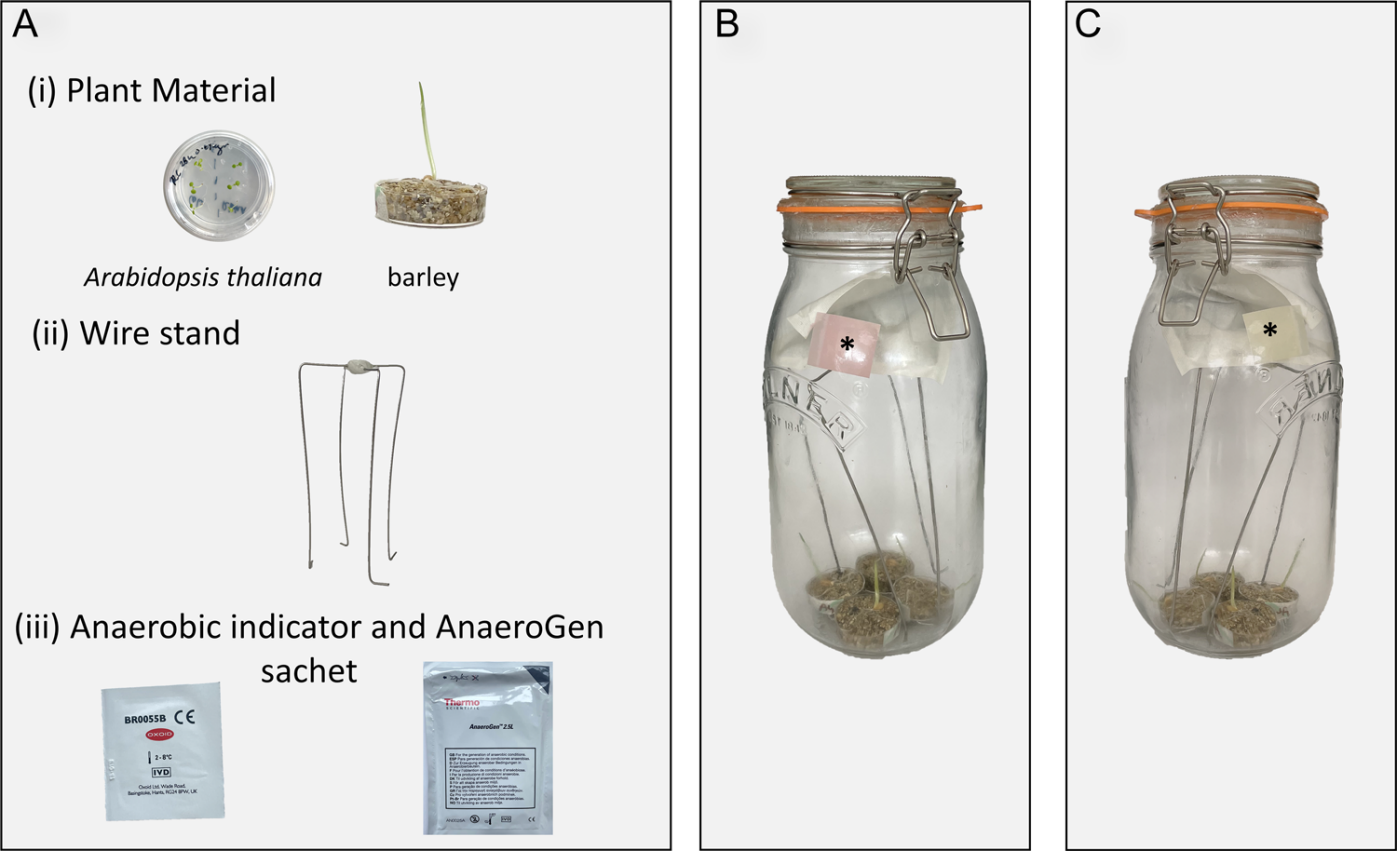
Outline of the developed protocol for anoxia treatment. (A) Place plant material (e.g., *Arabidopsis thaliana* or barley seedlings) on 35‐mm Petri dishes, wire stand, anaerobic indicator, and AnaeroGen sachet in the 2‐L Kilner jar (25.5 mm × 13.7 mm × 12.4 mm). (B) Close the jar 45–60 s after the AnaeroGen sachet has been opened. (C) As anoxia develops, the anaerobic indicator (*) will change from pink to white.

Anoxia treatment was performed in 2‐L airtight round Kilner Clip Top jars with rubber seals (product code 0025.493; Rayware Group, Liverpool, United Kingdom) purchased from a local Homebase Ltd. store (Dublin, Ireland). Galvanized 1.5‐mm steel wire and duct tape were used to construct simple stands (see Figure 1A for an example) for placing the Oxoid AnaeroGen 2.5‐L (AN0025, Thermo Fisher Scientific) sachets at the top part of the jar. Before the treatment, a thin layer of petroleum jelly was applied to the rubber seals of the jar to ensure an airtight lock. The Oxoid Resazurin Anaerobic Indicator (BR0055B, Thermo Fisher Scientific) was placed on the wall of the jar following manufacturer's instructions. To initiate anoxia treatment, the AnaeroGen foil sachets were opened and the AnaeroGen paper sachets were folded in half and immediately placed on the wire stand inside the Kilner jars. (See Appendix 1, section 1.1, for a complete list of required materials.) The jars were closed 45–60 s after opening the AnaeroGen foil sachets. AnaeroGen paper sachets contain ascorbic acid and activated carbon, which react with air, rapidly absorbing oxygen and producing carbon dioxide (Thermo Fisher Scientific, 2022). The Anaerobic Indicator consists of a cotton strip impregnated with pink resazurin solution that is reversibly reduced to colorless dihydroresorufin when oxygen becomes limited. The gradual change of the Anaerobic Indicator cotton strip from pink to white confirmed generation of an anoxic atmosphere within 2 h (Figure 1B, C). As the AnaeroGen sachets generate some heat due to an exothermic reaction between the active component ascorbic acid and oxygen, the sachets were also placed in control jars, but without an airtight seal, to ensure that the observed results of the anoxia treatment were not caused by an increase in temperature. See Appendix 2, section 2.1, for a detailed summary of the experimental protocol.

### Plant material and growth conditions

#### Arabidopsis thaliana

*Arabidopsis thaliana* wild‐type (Col0) and *anac017‐1* homozygous T‐DNA insertion knockout line were used for experiments. *Anac017* was a kind gift from Jim Whelan (LaTrobe University, Australia), derived from a T‐DNA insertion line originally obtained from the European Arabidopsis Stock Centre, generated by the Salk Institute Genomic Analysis Laboratory (Alonso et al., 2003) and previously characterized by Ng et al. (2013). Seeds were surface sterilized for 20 min in 20% (v/v) commercial bleach (final concentration of NaOCl approximately 1%), washed four times with sterile distilled water, and plated in 35‐mm Petri dishes containing half‐strength Murashige and Skoog basal salt medium (2.15 g L^−1^, M0221.0050; Duchefa Biochemie, Haarlem, The Netherlands), 0.6% agar (A1296; Sigma Aldrich, Darmstadt, Germany), supplemented with sucrose (S5391, Sigma Aldrich) or glucose (G0802.1000, Duchefa Biochemie) at concentrations of 30 mM or 90 mM (pH 5.6–5.8) (see below). The dishes were sealed with Leukopor tape (02453‐00; Essity, Hull, United Kingdom) that allows gaseous exchange but prevents moisture loss and placed at 4°C in the dark for 3 d before being moved to a 22°C constant temperature growth room for germination at the indicated light regime. See Appendix 1, section 1.2, for a list of required materials for growth of *A. thaliana* seedlings and Appendix 2, section 2.2, for protocol details.

#### Barley

Seeds of winter barley cultivars (Cavalier and Siberia) were a kind gift from Dr. Susanne Barth (Teagasc, Carlow, Ireland). The seeds were surface sterilized using a 20% commercial bleach (v/v) solution for 10 min, washed at least four times with sterile distilled water to remove any bleach residue, and placed in 90‐mm Petri dishes lined with moistened 85‐mm filter paper (approximately 20 seeds per Petri dish). The Petri dishes were sealed with Leukopor tape and placed at 4°C in the dark for 7 d to achieve synchronized seed germination. The seeds were allowed to germinate under a 16 h light/8 h dark regime at 22°C for 3 d before seedlings were used for experiments. See Appendix 1, section 1.2, for a list of required materials for growth of barely seedlings and Appendix 2, section 2.2, for protocol details.

### Anoxia treatment and recovery

#### Arabidopsis thaliana

**Figure 2:**
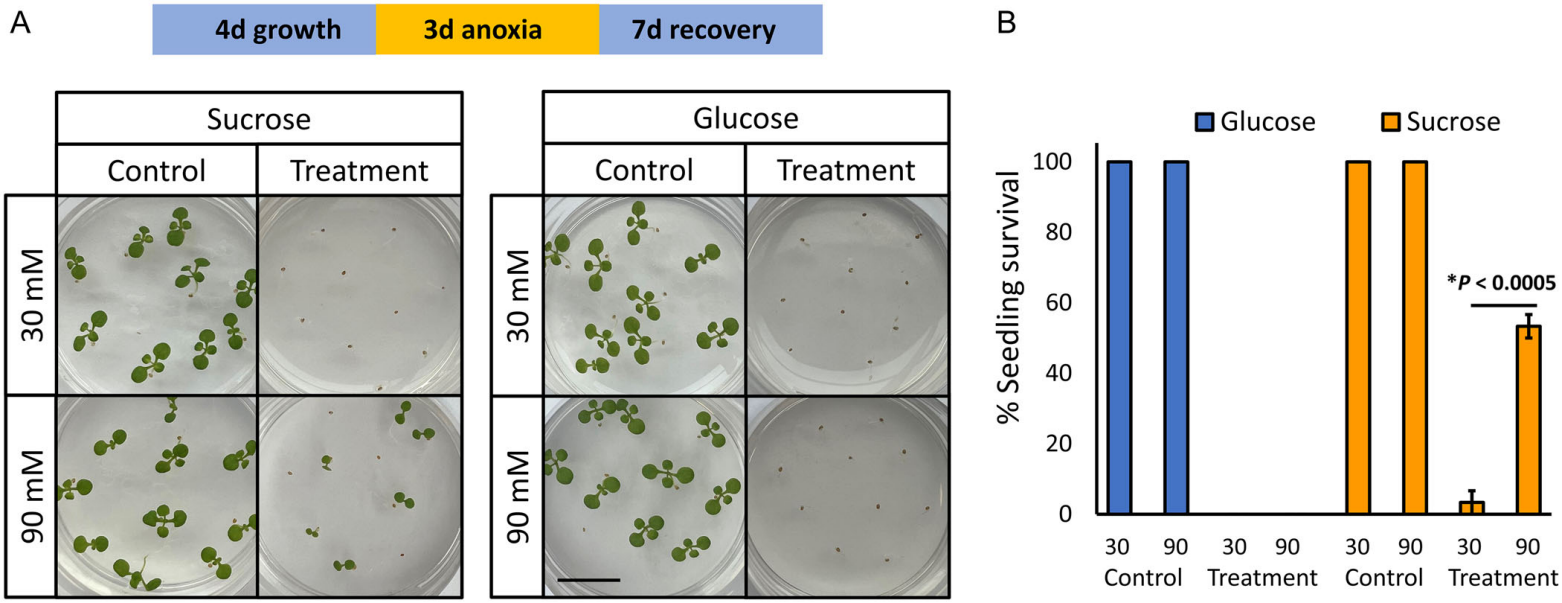
Protective effect of sucrose, but not glucose, on anoxia survival in *Arabidopsis thaliana*. Col0 seedlings were treated according to a protocol adapted from Banti et al. (2008). Seeds were germinated in the dark on 35‐mm Petri dishes supplemented with 30/90 mM of sucrose or glucose, subjected to a 3‐d anoxia treatment in the dark, followed by a 7‐d recovery period in 16 h light/8 h dark conditions. (A) Representative images of plants after the 7‐d recovery period. (B) Graph representing mean seedling survival (±SEM) at 30/90 mM treatments. Three experimental repeats (*n* = 10) were performed; *P* value obtained using Student's *t*‐test is indicated. Scale bar = 1 cm.

**Figure 3:**
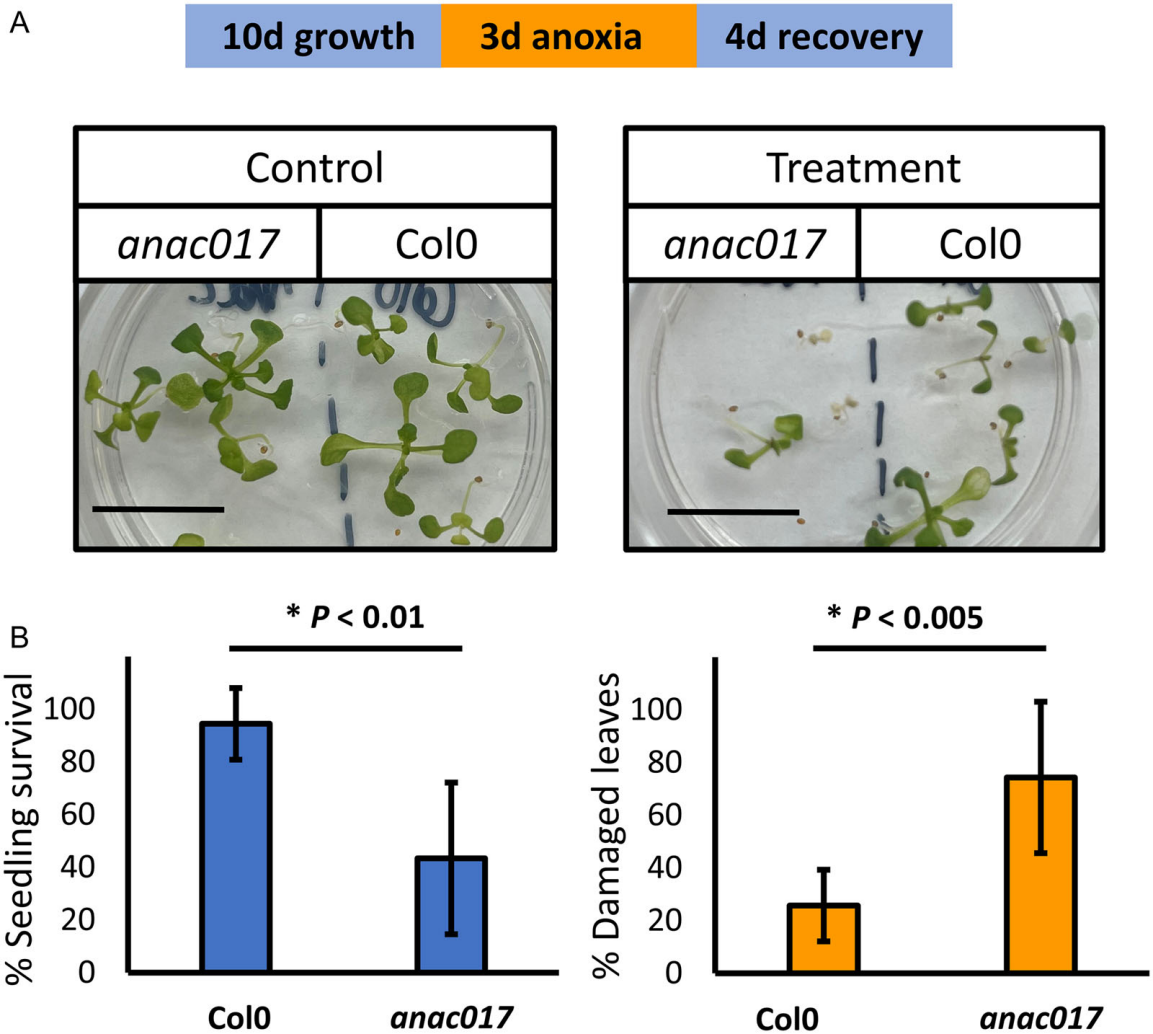
The *anac017* mutant shows impaired anoxia tolerance. Col0 and *anac017* seeds were germinated in 16 h light/8 h dark at 22°C on 35‐mm Petri dishes supplemented with 1% sucrose. Ten‐day‐old plants were subjected to a 3‐d anoxia treatment, followed by a 4‐d recovery period. Subsequently, plant survival and percentage of damaged leaves per seedling were recorded. (A) Representative images of Col0 and *anac017* plants after a 4‐d recovery period. (B) Graphs representing mean seedling survival and percentage of damaged leaves (±SEM). Six experimental repeats (*n* = 5) were performed. Untreated (control) seedlings displayed 100% survival and no leaf damage. *P* values indicate statistically significant difference between genotypes (Student's *t*‐test). Scale bar = 1 cm.

Four dishes with *A. thaliana* seedings were placed inside each Kilner jar, with Leukopor tape removed, for a 3‐d (72‐h) anoxia treatment. The anoxia treatment was interrupted after 72 h by opening the jars; the dishes were then resealed with Leukopor tape and the seedlings allowed to recover. The seedling age, light regime, and recovery time for the individual experiments are indicated in Figures 2 and 3, and the protocol details are provided in Appendix 2, section 2.2.

#### Barley

The 3‐d‐old barley seedlings were transferred to 35‐mm Petri dishes filled with soil substitute vermiculite saturated with distilled water. One seedling was placed per Petri dish, and four Petri dishes were placed inside each Kilner jar (two Petri dishes with each cultivar). The jars were closed and placed in the growth room for 24 h under a 16 h light/8 h dark regime at 22°C. On the following day, the jars were opened, seedling shoot length was recorded (Day 0, prior to anoxia treatment), and anoxia treatment was initiated. The anoxia treatment was interrupted after 24 h by opening the jars to allow re‐oxygenation by ambient air. The shoot growth of individual seedlings was measured immediately after the end of anoxia treatment, as well as after the first and second day of recovery. The details of the experimental protocol are summarized in Appendix 2, section 2.2. The samples of root tissue (approximately 50 mg fresh weight) were collected from the seedlings immediately after anoxia treatment for quantifying gene expression of *PDC* (pyruvate decarboxylase) and *ACO* (1‐amino cyclopropane 1‐carboxylic acid oxidase). The total RNA was isolated using an RNeasy Mini Plant Kit (Qiagen, Hilden, Germany) with the on‐column DNase I digestion step included. The RNA concentration and purity were measured using a NanoDrop Spectrophotometer (Thermo Fisher Scientific), and complementary DNA (cDNA) was synthesized using the RevertAid First Strand cDNA Synthesis Kit (Thermo Fisher Scientific). Quantitative PCR (qPCR) was performed using KAPA SYBR FAST qPCR Master Mix (KAPA Biosystems, Wilmington, Massachusetts, USA) in the ViiA 7 qPCR system (Thermo Fisher Scientific) using protocol and primer sequences described by Luan et al. (2018).

#### Statistical analyses

The obtained data were analyzed (Student's *t*‐test) using IBM SPSS Statistics for Windows software (version 27.0; IBM, Armonk, New York, USA).

### Applicability of the airtight jar method for studying anoxia responses in the model species *Arabidopsis thaliana*

We used the developed method to successfully replicate the results from a previous study that utilized an enclosed anaerobic workstation for investigating anoxia tolerance in this crucial model species (Banti et al., 2008). Banti et al. (2008) reported a protective effect of exogenous sucrose, but not glucose, on anoxia tolerance in *A. thaliana*. We adapted their protocol to the airtight jar method to confirm that similar results can be obtained without the use of an anaerobic workstation. Indeed, anoxia treatment (72 h) of 4‐d‐old, dark‐germinated Col0 seedlings resulted in no survival on solid medium supplemented with 30 mM sucrose, in contrast to 53% survival on medium supplemented with 90 mM sucrose (Figure 2). However, a similar effect has not been observed when the growth medium was supplemented with glucose, with no seedlings surviving anoxia treatment at both 30 mM and 90 mM concentration, also in agreement with Banti et al. (2008). In addition, we used the developed method to estimate the anoxia tolerance of mitochondrial retrograde signaling mutant, *anac017*, that has been previously reported to show impaired submergence tolerance (Bui et al., 2020; Meng et al., 2020) (Figure 3). For these experiments, plants were grown for 10 d (16 h light/8 h dark) prior to anoxia treatment, as the submergence sensitivity phenotype of *anac017* has been reported to be enhanced with seedling age (Bui et al., 2020). As anticipated, treatment of Col0 and *anac017* plants revealed higher anoxia sensitivity of *anac017* seedlings, which showed significantly lower treatment survival and an increased level of leaf damage (Figure 3). These results highlight that the airtight jar method offers a simple protocol to study the effect of growth conditions and genotype on anoxia tolerance in the model species *A. thaliana* and can be easily adapted to meet experimental requirements (e.g., in terms of age of treated plants, chemical supplementation, light and temperature regime).

### Applicability of the airtight jar method for studying anoxia responses in barley

**Figure 4:**
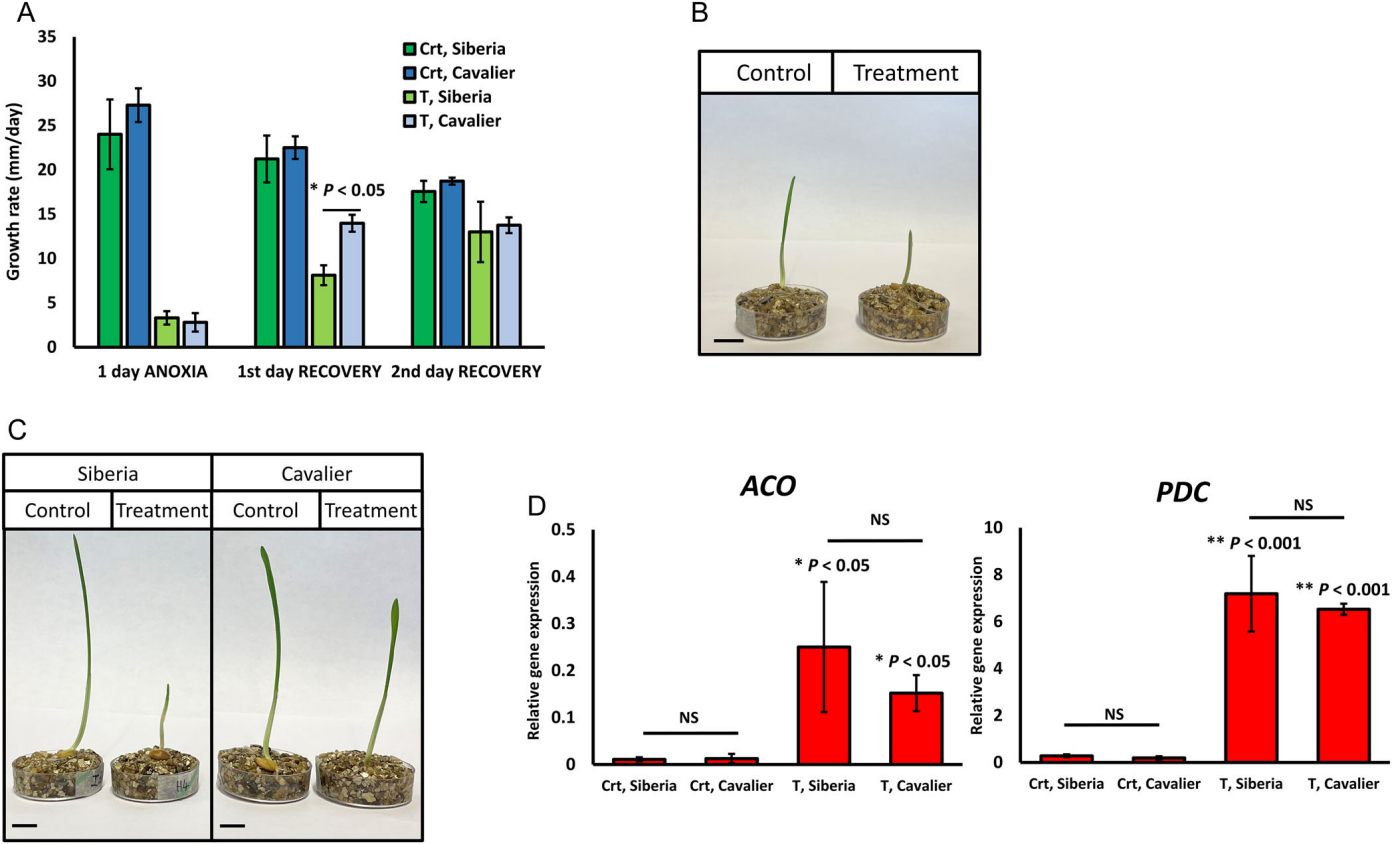
Response of barley seedlings to anoxia treatment. Cavalier and Siberia seeds were germinated in 16 h light/8 h dark at 22°C on 85‐mm moistened filter paper inside 90‐mm Petri dishes. Four‐day‐old plants were subjected to a 24‐h anoxia treatment, followed by a 48‐h recovery period. (A) Barley shoot growth rate (mm/day) during the anoxia treatment and recovery period. Cavalier and Siberia, two winter barley cultivars, were tested. Bars represent the mean of three replicates (±SEM) (*n* = 8). Crt = control, T = Treatment; *P* value determined using Student's *t*‐test is indicated. (B) A 24‐h anoxia treatment induces shoot growth cessation in barley seedlings. Scale bar = 1 cm. (C) Cavalier shows faster shoot growth recovery following the treatment; control and treated Siberia and Cavalier seedlings after a 24‐h anoxia treatment plus a 24‐h recovery period are shown. Scale bar = 1 cm. (D) Expression of *ACO* and *PDC* genes induced by the 24‐h anoxia treatment in both cultivars. Bars represent the mean of three experimental replicates (±SEM) (*n* = 2). Indicated *P* values were determined using Student's *t*‐test on log_2_‐transformed relative gene expression data. NS = not significant.

Barley is relatively sensitive to flooding and soil waterlogging (Setter and Waters, 2003; de San Celedonio et al., 2014). The identification of barley germplasm with increased tolerance to oxygen deprivation, and characterization of the genetic mechanisms underlying this tolerance, is therefore urgently required. Herein, we tested the developed protocol on two winter barley cultivars, Cavalier and Siberia (Figure 4). As expected, the anoxia treatment resulted in almost complete shoot growth cessation in both cultivars (Figure 4A). Shoot growth resumed during an anoxia recovery period, with Cavalier showing faster shoot growth recovery compared to Siberia. This is in line with a recently published study where prolonged (15 d) waterlogging treatment, followed by six weeks of recovery, significantly reduced the plant height of Siberia but had no effect on the Cavalier cultivar (Miricescu et al., 2021). Moreover, *ACO*, the gene involved in ethylene biosynthesis (Houben and Van de Poel, 2019), and *PDC*, which catalyzes the first step in the ethanolic fermentation pathway (Jackson and Colmer, 2005), were significantly induced by the anoxia treatment in the roots of both cultivars (Figure 4D). Previously, upregulation of these genes in barley root tissue was suggested to be a critical factor driving the flooding responses in this species (Luan et al., 2018). Additionally, during optimization of the protocol for barley we also confirmed that there was no growth rate reduction when AnaeroGen sachets were placed in the jars without an airtight seal (compared to jars without sachets), which further suggested that the effects of the applied treatment were not due to the generated heat but resulted from oxygen deprivation.

Collectively, the results suggest that the developed protocol for anoxia treatment: (i) facilitates imposing rapid and severe oxygen deprivation stress on crop seedlings in a repetitive and reliable manner, (ii) allows quick detection of differences between studied cultivars that mirror those observed in other experimental systems, and (iii) induces a gene expression profile typical for response to low oxygen conditions. Consequently, the airtight jar method presented in this study has the potential to advance research on the mechanisms underlying responses to oxygen deprivation at an early plant growth stage, as well as the identification of tolerant germplasm, in barley and other crop species. It needs to be highlighted that while the method can be used for a variety of species, the size of the airtight Kilner jars makes the protocol suitable for studying anoxia responses in small species, or in plants at early developmental stages, such as barley seedlings, as described herein.

## CONCLUSIONS

We developed a novel protocol for studying anoxia tolerance in plants using airtight Kilner jars and Oxoid AnaeroGen anaerobic gas‐generating sachets. The low cost, simplicity, and adaptability of the proposed experimental setup suggest that it would be of interest to researchers studying oxygen deprivation responses in plants who have no access to anaerobic workstations. One consideration while using the developed protocol is that when an AnaeroGen sachet is placed in an airtight jar, the oxygen is rapidly absorbed, leading to simultaneous generation of carbon dioxide, with resulting CO_2_ levels of 9–13% (ThermoFisher Scientific, 2022). Similarly, CO_2_ is found to accumulate in waterlogged soils (Setter and Waters, 2003), and toxic concentrations of CO_2_ in soil were shown to negatively affect plant growth in previous studies. For example, prolonged exposure to soil CO_2_ concentrations of 41–65% reduced root water absorption, chlorophyll, starch content, and total biomass (He et al., 2019). It is therefore possible that the effects observed here are partially linked to the high CO_2_ levels generated in the jars. This increase in CO_2_ concentration needs to be taken into account when planning experiments involving the airtight jar method, especially when prolonged anoxia treatment is applied. In the future, additional optimizations of the method that could potentially reduce CO_2_ accumulation in the jars are possible, for example, based on absorption of CO_2_ by dry soda lime.

In this study, we demonstrated that the established airtight jar method is suitable for studying the effect of growth conditions and genotype on anoxia tolerance in the model species *A. thaliana*. Herein, we applied anoxia treatments of up to 72 h, but if required this period could be extended, with the used resazurin indicator making it possible to monitor continued maintenance of anaerobic conditions. Moreover, the method is transferable between species and can be used to study anoxia‐induced physiological and molecular responses, as well as to identify differences in cultivar tolerance in crops such as barley. In conclusion, the described method has a strong potential to advance both basic and applied research and is particularly relevant considering the urgent need to increase our understanding of how plants cope with oxygen deprivation stress.

## AUTHOR CONTRIBUTIONS

J.K. conceived an original idea for the method that was further developed with all authors (O.L.S., R.B., S.B., P.F.M.). O.L.S., R.C., and S.B. performed the experiments. J.K. and O.L.S. drafted the initial version of the manuscript. All authors contributed to the preparation of the manuscript and approved the final version.

## APPENDIX 1 Materials required for use with the anoxia treatment protocol and plant material growth.

NOTE: Materials and equipment indicated here have been used in our laboratory. Substitutions with equivalent materials can be carried out as required.

**1.1 Anoxia treatment**

1. 2‐L airtight round Kilner Clip Top jar with rubber seal (product code 0025.493; Rayware Group, Liverpool, United Kingdom)
2. Galvanized 1.5‐mm steel wire
3. Pliers
4. Duct tape
5. Petroleum jelly
6. Oxoid Resazurin Anaerobic Indicator (BR0055B; Thermo Fisher Scientific, Basingstoke, United Kingdom)
7. Oxoid AnaeroGen 2.5‐L Sachet (AN0025A; Thermo Fisher Scientific)

**1.2 Growth of plant material for anoxia treatment**

***Arabidopsis thaliana—***

1. Commercial bleach
2. Sterile distilled water
3. Murashige and Skoog basal salt mixture (M0221.0050; Duchefa Biochemie, Haarlem, The Netherlands)
4. Agar (A1296; Sigma Aldrich, Darmstadt, Germany)
5. Sucrose (S5391; Sigma Aldrich)
6. Dishes (35‐mm Petri dishes)
7. Micropipette and tips
8. Barky Ultipette and tip (PS‐10; Barky Instruments International, Kent, United Kingdom)
9. Leukopor tape (1.25 cm width) (02453‐00; Essity, Hull, United Kingdom)

***Hordeum vulgare*** **(barley)** ***—***

1. Commercial bleach
2. Distilled water
3. Dishes (35‐mm, 90‐mm Petri dishes)
4. Filter paper (85 mm)
5. Forceps
6. Leukopor tape (1.25 cm width) (02453‐00; Essity)
7. Vermiculite

## APPENDIX 2 A detailed summary of the anoxia treatment protocol and plant growth conditions.

**2.1 Detailed anoxia jar protocol for anoxia tolerance assay**

1. Place plant material (see section 2.2. for details) into the bottom of a Kilner jar. Insert an in‐house, custom‐built wire support into each jar, taking care not to tip over the plants.
  NOTE: The wire support is made by cutting two equal lengths of 1.5‐mm galvanized steel wire using a pliers. The wire lengths are then molded into two flat‐bottomed U‐shapes. The U‐shaped wire lengths are placed perpendicular to one another and held together with duct tape to form a platform of approximately equal height to the Kilner jar being used for anoxia treatment (Figure 1A).
2. Place a thin coat of petroleum jelly on both sides of the rubber seal, the glass lid, and the body of the jar where the seal is formed. This facilitates the formation of an airtight seal and anoxic atmosphere inside the jar during anoxia treatment.
3. Open an Oxoid Resazurin Anaerobic Indicator strip packet and immediately place the strip onto the glass inside the jar where it is visible (Figure 1B). The indicator will change color to red/pink to indicate the presence of oxygen.
4. Immediately following indicator strip insertion, open the Oxoid AnaeroGen 2.5‐L sachet packaging, fold the sachet in half, and place onto the wire support inside the jar. Close the jar within 45–60 s after opening the AnaeroGen packaging (Figure 1B indicates experimental setup) and place under the desired growth conditions. Once the anoxic atmosphere has been generated inside the jar, the indicator paper will turn white; this typically takes up to 2 h in the proposed setup (Figure 1C).
  NOTE: Ensure that the edges of the AnaeroGen sachet are not caught in the lid of the Kilner jar as this will impede the airtight seal formation.
  NOTE: The Oxoid AnaeroGen 2.5‐L sachets generate some heat (exothermic reaction) during oxygen absorption. To ensure the anoxia treatment results were not caused by the indirect effect of this reaction, the control jars containing the wire structure with an AnaeroGen sachet were left unsealed.
5. Following the desired anoxia treatment period, open the jar and allow ambient air to enter the jar. The oxygen indicator paper should return to a red/pink color in <1 min, indicating the presence of oxygen and cessation of anoxia treatment.

**2.2 Plant material growth conditions and species‐specific considerations for anoxia jar treatment**

***Arabidopsis thaliana*** **—**

NOTE: *Arabidopsis thaliana* seed sterilization and plating was carried out in sterile conditions using a laminar flow hood.

1. Surface sterilize the *A. thaliana* seeds in 20% commercial bleach (v/v) for 20 min, ensuring a final concentration of approximately 1% NaOCl.
2. Rinse the seeds with sterile distilled water four times.
3. Using an Ultipette, plate *A. thaliana* seeds onto half‐strength Murashige and Skoog basal salt mixture (2.15 g L^−1^) medium, 0.6% agar supplemented with 1% sucrose (pH 5.6–5.8) in 35‐mm Petri dishes.
4. Seal dishes with Leukopor tape, invert, and place in the dark at 4°C for 3 d. Leukopor tape is used in place of parafilm as it allows gaseous exchange while preventing moisture loss.
5. Transfer dishes to a growth chamber with the desired growth conditions prior to anoxia treatment.
6. Once seedlings are at the desired growth stage, remove the Leukopor tape from the *A. thaliana* seedling dishes and place four dishes into the Kilner jar.
  NOTE: A 30‐cm forceps may be used for placing dishes with seedlings inside the jar.
7. Proceed with section 2.1 (“Detailed anoxia jar protocol for anoxia tolerance assay”).
8. Following anoxia treatment, remove the dishes from the Kilner jar and reseal the dishes using Leukopor tape to prevent contamination and moisture loss occurring during a recovery timeframe.

***Hordeum vulgare*** **(barley)—**

1. Surface sterilize the barley seeds in 20% commercial bleach (v/v) for 10 min. This ensures a final concentration of approximately 1% NaOCl.
2. Rinse the seeds in distilled water 6–8 times.
3. Place one 85‐mm filter paper sheet inside a 90‐mm Petri dish and moisten with 6 mL of distilled water.
4. Place approximately 20 seeds on the filter paper in each dish with forceps and seal with Leukopor tape.
5. Place the dishes at 4°C in the dark for 7 d for stratification and vernalization.
6. Transfer the dishes to a growth chamber with desired growth conditions prior to being utilized in anoxia tolerance experiments.
7. Once the seedlings reach the desired growth stage, soak vermiculite with distilled water and leave to sit for 15 min. Fill 35‐mm Petri dishes completely with moist vermiculite, leveling off any excess to achieve a flat surface.
8. Using forceps, transfer barley seedlings that have a visible root and shoot system from the 90‐mm Petri dishes onto the surface of the vermiculite. Place one seedling per 35‐mm Petri dish. Press the seedling down gently to ensure surface contact between the seedling roots and vermiculite.
9. Place four 35‐mm Petri dishes into each Kilner jar and seal the jar to prevent drying out of vermiculite dishes.
10. Place the Kilner jars into a growth chamber with the desired growth conditions for 24 h prior to anoxia treatment.
11. Proceed with section 2.1 (“Detailed anoxia jar protocol for anoxia tolerance assay”).
